# Through-Process Finite Element Modeling for Warm Flanging Process of Large-Diameter Aluminum Alloy Shell of Gas Insulated (Metal-Enclosed) Switchgear

**DOI:** 10.3390/ma12111784

**Published:** 2019-06-01

**Authors:** Da-Wei Zhang, Tian-Lin Shi, Sheng-Dun Zhao

**Affiliations:** School of Mechanical Engineering, Xi’an Jiaotong University, Xi’an 710049, China; stl123@stu.xjtu.edu.cn (T.-L.S.); sdzhao@mail.xjtu.edu.cn (S.-D.Z.)

**Keywords:** local heating, warm flanging, finite element modeling, large-diameter shell, gas insulated (metal-enclosed) switchgear, forming defects, temperature field

## Abstract

The large diameter metal shell component (LDMSC) is an important part of gas insulated (metal-enclosed) switchgear (GIS). The LDMSC with multi branches is filled with gas under certain pressure. The plastic forming process is an efficient approach to manufacturing the high reliability LDMSC. The warm flanging process has been widely used to form LDMSC using aluminum alloy. The forming process is characterized by local heating, and the distribution of temperature is strongly inhomogeneous. Although the wall thickness of the shell is 10 mm to 20 mm, the ratio of outer diameter to thickness is more than 40. These present some difficulties in the flanging process and result in some forming defects. Detailed forming characteristics are hard to obtain by analytical and experimental methods. Thus, the through-process finite element (FE) modeling considering heating, forming, unloading, and cooling is one of the key problems to research the manufacturing process of LDMSC. In this study, the through-process FE modeling of the warm flanging process of LDMSC using aluminum alloy was carried out based on the FORGE. The thermo-mechanical coupled finite element method was adopted in the modeling, and the deformation of the workpiece and the die stress were considered together in the modeling. A full three-dimensional (3D) geometry was modeled due to inhomogeneous distribution in all directions for the temperature field. The simulation data of local flame heating could be transferred seamlessly to the simulations of the deforming process, the unloading process, and the cooling process in the through-process FE model. The model was validated by comparison with geometric shapes and forming defects obtained from the experiment. The developed FE model could describe the inhomogeneous temperature field along circumferential, radial, and axial directions for the formed branch as well as the deformation characteristic and the unloading behavior during the warm flanging process. By using the FE model, the forming defects during the flanging process and their controlling characteristics were explored, the evolution of the temperature field through the whole process was studied, and deformation and springback characteristics were analyzed. The results of this study provide a basis for investigating deformation mechanisms, optimizing processes, and determining parameters in the warm flanging process of a large-diameter aluminum alloy shell component.

## 1. Introduction

**Figure 1:**
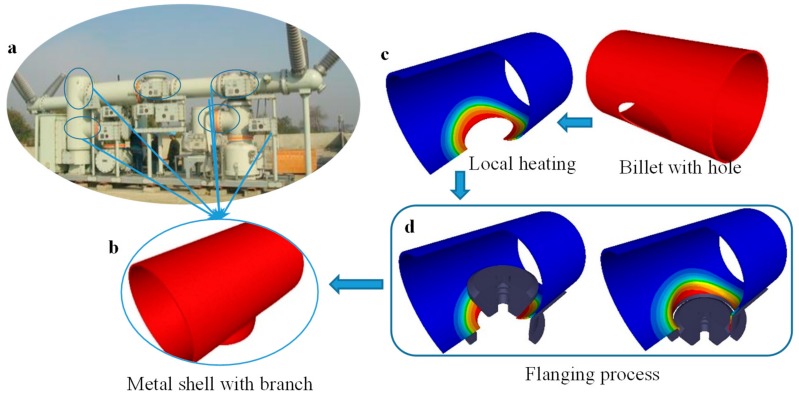
Metal shell of gas insulated (metal-enclosed) switchgear (GIS) and the warm flanging process: (**a**) representative GIS; (**b**) metal shell with branch; (**c**) local loading; and (**d)** flanging process for warm flanging.

Gas insulated (metal-enclosed) switchgear (GIS) technology has been widely used in electrical power systems [1,2,3,4]. For GIS, as shown in Figure 1a, the circuit breaker, the isolating switch, the earthing switch, the transformer, and other control and protective devices are concentrated in an enclosed metal shell. The metal shell with multi branches has a large physical dimension and works with compressed gas, such as SF_6_ gas. The forming quality of the branches that form large diameter tubes play an important role in the assembly time and the seal ability. Thus, the manufacture of large diameter metal shell components (LDMSC) is a key link for GIS.

The plastic forming process is an efficient approach to manufacturing the high reliability LDMSC. The warm flanging process has been widely used to form LDMSC using aluminum alloy. Although the wall thickness of the shell is often 10 mm to 20 mm, the maximum outer diameter is more than 1000 mm, and the ratio of outer diameter to thickness is more than 40—sometimes even more than 50. It has thin-walled tube characteristics, but the deformation presents bulk forming characteristics. Furthermore, the forming process is characterized by local heating, as shown in Figure 1c, and the distribution of temperature is strongly inhomogeneous. These present some difficulties in the flanging process and result in some forming defects. Analytical and experimental detailed forming characteristics are hard to obtain.

The sheet metal flanging processes under room temperature, such as stretch flanging, shrink flanging, shrink curved flanging, etc., have been extensively studied. Analytical and experimental studies were the main research method for the flanging process twenty years ago. Analytical study on stress and strain distributions and the flange length in the hole flanging process were carried out by Wang and Wenner [5]. Li et al. [6] studied the characteristics of the deformation, the stress properties, the strain distribution, and various factors influencing the deformation in shrink curved flanging based on experiments. Wang et al. [7] established an approximate theory and failure criteria (fracturing and wrinkling) for two basic flanging operations—shrink and stretch flanging. Hu et al. [8] presented two modified analytical models for stretch flanging and shrink flanging based on the assumption of total plasticity theory and membrane strain.

Numerical methods have been widely adopted in the analysis of metal forming processes [9,10,11,12]. Feng et al. [13] investigated the effects of geometrical parameters on the formability of stretch curved flanging by using the finite element method (FEM). The FEM was also used to study the formability limitation during the hole flanging process by Huang and Chien [14]. A two-dimensional (2D) axisymmetric model was also developed to evaluate the formability of an aluminum alloy sheet during the hole flanging process [15]. Generally, an axisymmetric model is adopted in the finite element (FE) model of the hole flanging process due to the axial symmetry of the round hole. Chen [16] developed a three-dimensional (3D)-FE model to study the formability limitation during the elliptic hole flanging process, where only quarter geometry of the workpiece and the dies was adopted according to the symmetry. The 3D-FE model was developed by Yu et al. to study the elliptic hole flanging for advanced high-strength steels [17]. A 3D-FE model was also used to study the thickness distribution of hole flanging by different punch geometries [18]. The thicknesses of the sheets were all less than 2 mm, and the shell-element type was adopted in the above studies. The flanging process is always studied by regarding the plate as a sheet, thus the research can focus on the neutral surface and ignore the thickness. The shell element adopted in FE modeling can describe the deformation characteristic during hole flanging with thin sheets [19]. However, this method is not proper for the thick plate, especially the thick cylinder, since the shape is influenced by the deformation along the thickness direction.

Numerical and experimental studies on hole flanging with the thickness of 3–10 mm were carried out by Kumagai and Saiki [20], where a 2D-FE model was used for numerical study. In order to obtain a substantial flange from a thick plate with the thickness of 5 mm, an upsetting flanging process (hole flanging combined with upsetting) was developed by Lin et al. [21], where a 3D numerical simulation based on DEFORM code was used to study the influence of geometric parameters in the process, and a quarter FE model was adopted (although the process was an axisymmetric problem). By using Dynaform code, the influence of geometric parameters on the cold flanging for aluminum alloy 5754 T-pipe with a thickness of 10 mm and an outer diameter of 356 mm was studied by Ma et al. [22]. However, using the shell-element type to describe the severe deformation of the workpiece with a thickness of 10 mm is not very reasonable.

The thermo-mechanical coupled problem was not considered in the above research due to the cold forming process. Generally, warm or hot forming will be adopted in industrial production for flanging with thicknesses more than 10 mm. An and Liu [23] analyzed the hot hole flanging process of a connection tube in a thick-wall head by using DEFORM code. However, the ratio of outer diameter to thickness was about 11.7 in the study [23], and it showed typical thick-walled tube characteristics. In the present study, the ratio of outer diameter to thickness was more than 40—sometimes even more than 50—and it had notable thin-walled tube characteristics. Furthermore, the maximum thickness in the present study was 20 mm, and it was much less than the thickness (300 mm) in the study [23]. The local heating characteristics are not presented, as they were not considered in the FE modeling in the study [23].

The multi-pass hot flanging process of an X80 steel pipe with a thickness of 18.4 mm and an outer diameter of 508 mm was analyzed by numerical simulation [24]. The whole billet was heated in the study. This method is not fit for the flanging process of large-diameter pipes. The modeling method for the initial temperature filed in the study [24] did not describe the actual heating process for the flanging process of a large-diameter pipe. The local heating and warm flanging FE model of the aluminum alloy 5083 shell of GIS was developed by Ben et al. [25]. However, the quarter FE model was adopted, and the circumferential area of the elliptical prefabricated hole was heated at the same time. There was notable difference in the actual heating process by a flame gun.

The thermo-mechanical coupled FE model was also used in the analysis of warm or hot stamping. A thermo-mechanical coupled FE model considering the failure of welding spots was developed to analyze the hot stamping process of B1500HS steel [26]. The thermo-mechanical coupled FEM was also used to analyze the warm stamping of the Ti-6Al-4V sheet [27]. The thicknesses of the sheets used in above studies were both less than 2 mm, and shell elements were used to mesh the sheets. The 2D plane strain solid element was used to describe the sheet in the numerical simulation of the hot stamping process [28], where the thickness of the sheet was 2 mm. However, the modeling method is not proper for the flanging process in this study due to severe plastic deformation and the 3D problem.

The warm flanging process of LDMSC combines the forming characteristics of sheet forming and bulk forming. The existing knowledge about the flanging process to form sheet forming or bulk forming does not include the warm flanging process of LDMSC. Analytical and experimental detailed forming characteristics are hard to obtain due to the inhomogeneous temperature and the complicated deformation. The quarter FE model (developed in the study [25]) with simultaneous heating along the circumferential direction of the prefabricated hole cannot describe this process well. Thus, the through-process FE model considering heating and deforming was developed by the Transvalor FORGE NxT 2.1. (Transvalor, Biot, France) The thermo-mechanical coupled finite element method and the full geometry of dies and workpiece were adopted in the modeling, and the deformations of the workpiece and the die stress were considered together in the modeling. The circumferential and the radial inhomogeneity of the temperature field along the elliptical prefabricated hole were well considered in the developed full 3D-FE model. The simulation data of local flame heating could be transferred seamlessly to the simulation of the deforming process in the through-process FE model. The model was validated by comparison with geometric shapes and forming defects obtained from the experiment. The model provides a basis for investigating deformation mechanisms, optimizing processes, and determining parameters in the warm flanging process of LDMSC with aluminum alloy.

## 2. Description of Warm Flanging Process

The LDMSCs of GIS are T-branch pipes or pipes with multi branches, such as the physical photo shown in Figure 1a. The local heating and flanging processes were implemented to form one branch, as shown in Figure 1c,d. Only the local area around the elliptical prefabricated hole of the billet was heated. The area and the strength of the unheated area were much greater than the area and the strength of the heated area. The unheated area provided a strong restriction to the deformed area of the branch. Thus, the influence of deformation and temperature on the other area was little. In particular, in the manufacturing process of LDMSC with multi branches, the forming process of one branch has little influence on the forming processes of other branches.

**Figure 2:**
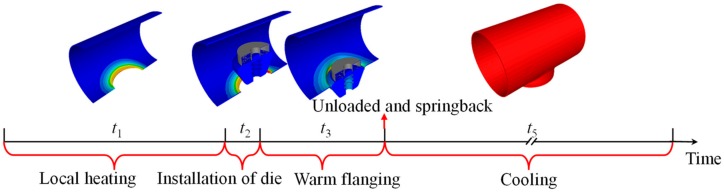
Through-process of heating-forming-cooling for manufacturing GIS shell.

One pass of the warm flanging process had five processing stages, as shown in Figure 2.

The first stage was the local heating process. In general, flame heating is adopted for LDMSC with aluminum alloy in China. The deforming area around the elliptic hole was heated to about 150–250 °C. After local heating, circumferential and radial temperature fields along the elliptical prefabricated hole were both inhomogeneous.

The second stage was the waiting process. At this stage, the dies were transferred and installed, and the billet waited and cooled.

The third stage was the warm flanging process. At this stage, the force field coupled with the temperature field.

The unload and springback process came after the flanging process. The springback was done instantaneously, thus the time of the fourth stage was considered as zero, i.e., *t*_4_ = 0 s.

The fifth stage was the cooling process. In general, the air cooling process is adopted. Thus, the time of the fifth stage was much greater than those of the above stages. 

The times of the five stages used in this study are listed in Table 1.

For the manufacture of LDMSC with multi branches, multi passes were implemented. Considering the small influence of deformation on the other area, a typical one-pass of the warm flanging process was chosen to investigate the FE modeling method in the present study. By changing the geometry model and the relative parameters and then repeating the heating-cooling-flanging-springback-cooling, the developed FE model in the present study was also suitable for the multi passes forming process of LDMSC with multi branches.

## 3. Through-Process Finite Element Modeling for Local Heating and Integrally Flanging Whole Process

Based on the FE soft environment of FORGE code, a thermo-mechanical FE model for a local heating-cooling-flanging-springback-cooling through-process was developed.

### 3.1. Material Parameters

The materials of LDMSC and flanging dies used in the present study were AA5083 aluminum alloy and H13 die steel, respectively. The die stress analysis was considered in the present study, thus the material properties of the billet and the dies were needed.

The mechanical property of the AA5083 aluminum alloy is related to the temperature and the strain rate. Thus, the Hansel–Spittel model was adopted to describe the mechanical property of the AA5083 aluminum alloy, which could describe the relationship between viscoplastic flow law and strain, strain rate, and temperature.

**Figure 3:**
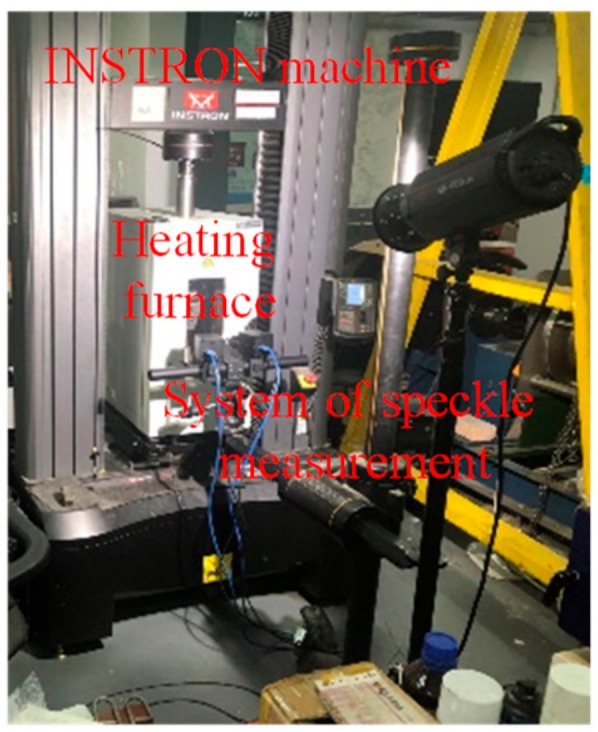
Uniaxial tension testing system.

The tension tests with large strain for the AA5083 aluminum alloy were carried out by the testing system (as shown in Figure 3) by combining the INSTRON machine and the XJTUDIC 3D measurement system. The relationships between stress and strain wihtin 20–250 °C and 0.001–0.1 s^−1^ were obtained. According to the experimental data, the parameters in the Hansel–Spittel model were determined, and thus the constitutive model (Equation (1)) of the AA5083 aluminum alloy was obtained.

(1) $$\sigma =2593.1535\varepsilon^{0.47685}{\dot{\varepsilon }}^{0.05893}{}^{+1.9097\times 10^{-4}T}\exp\left(\frac{8.60291\times 10^{-7}}{\varepsilon }-1.58328\times \varepsilon \right){\left(1+\varepsilon \right)}^{-0.0091T}exp\left(0.00292T\right)T^{-0.15383}$$

where ε is the strain; $\dot{\varepsilon }$ is the strain rate; *T* is the temperature during deformation.

The Hansel–Spittel model (Equation (2)) was also adopted to describe the mechanical property of the H13 die steel, where the parameters in the Hansel–Spittel model were taken from the database of FORGE code.

(2) $$\sigma =2821.246\exp\left(0.0029T\right)\varepsilon^{-0.10727}{\dot{\varepsilon }}^{0.13444}\exp\left(\frac{-0.0462}{\varepsilon }\right)$$

The elastic and the physical properties of the AA5083 aluminum alloy and the H13 die steel were determined according to the database of FORGE code, journal articles, and handbooks [25,29,30,31], as listed in Table 2.

### 3.2. Geometry Modeling and Meshing

The billet used in this study was a large tube with an elliptical prefabricated hole, where the thickness and the inner diameter of the tube were 18 mm and 1000 mm, respectively. The size of the prefabricated hole was a controllable parameter, and 695 × 430 mm was adopted in this section.

**Figure 4:**
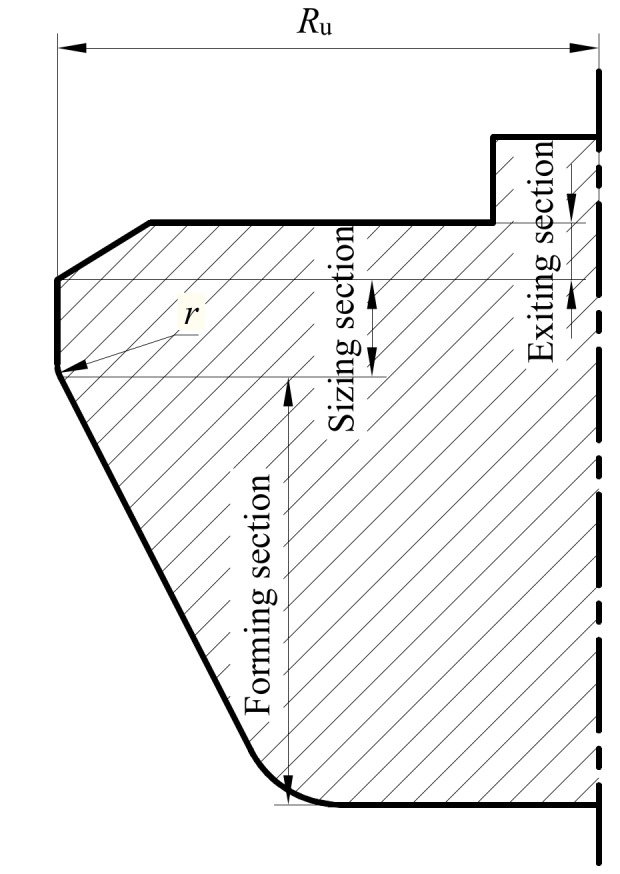
Axial section of upper die.

The upper die for the flanging process was a circular truncated cone with cone angle α, as shown in Figure 4. The upper die was divided into three sections—forming section, sizing section, and exiting section—along its axis. There was a transition fillet between the forming section and the sizing section, whose radius was *r*. α = 27°, and *r* = 20 mm was adopted in this section.

The lower die for the flanging process had a round hole to accommodate the formed branch, and the lower die was for supporting the shell of GIS. The diameter of the round hole of the lower die was 800 mm in this study.

**Figure 5:**
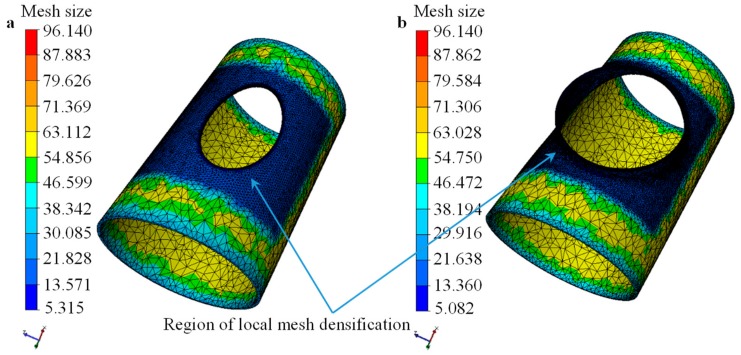
Distribution of mesh size for billet/workpiece: (**a**) initial mesh; (**b**) mesh after flanging process.

The tetrahedron elements were used to mesh the billet/workpiece and the dies, and the mesh local refinement was adopted for the initial mesh. The region around the elliptical prefabricated hole was the refining region, and the largest initial mesh size in this region was about 12 mm, which was 0.33% of the perimeter of the elliptical prefabricated hole, as shown in Figure 5a. The initial meshes for the upper die and the lower die were inhomogeneous, and the meshes in the zones contacted with the workpiece were fine.

In order to ensure the calculation accuracy under large deformation, local refine meshing, re-meshing, and adaptive meshing techniques were adopted in the simulation of the flanging process (i.e., third stage shown in Figure 2). The mesh for the severe plastic deformation region was finer due to re-meshing, as shown in Figure 5b, thus the number of elements for the workpiece increased from 49,138 to 93,377 during the flanging process. Then, the number of elements for the workpiece was a constant during the unloading and the cooling processes. The numbers of elements for the upper die or the lower die were a constant during the heating-flanging-cooling through-process.

A set of mesh for the billet/workpiece was used in the whole forming process. Thus, the thermal and the mechanical data could be transferred seamlessly from local heating to waiting (i.e., cooling), to flanging, to unloading, and then springback to cooling.

### 3.3. FE Modeling of Local Heating by Flame

**Figure 6:**
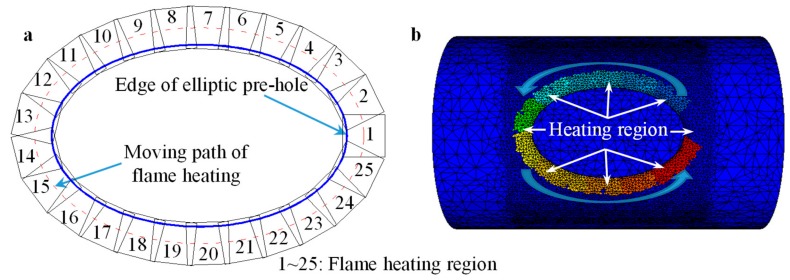
Local heating model: (**a**) heating path; (**b**) finite element (FE) model.

Before the flanging process, a flame gun traveled around the elliptical prefabricated hole to heat the forming region. In order to simulate the inhomogeneous heating process, a heating source was set to travel around the prefabricated hole. The moving path of the heating source was an ellipse concentric with the prefabricated hole, i.e., the moving path of the flame heating, as shown in Figure 6a, where the offset distance between the concentric ellipses was 40 mm. There were *n* same heating regions along the moving path of flame heating, and the distances between adjacent regions were the same. The size of the heating region was 100 × 100 mm, which was determined according to flame area from the flame gun; *n* = 25 was determined according to the geometry of the billet and the size of the heating source.

The local heating was simulated by the heat exchange between the billet/workpiece and the heating region. The influence of heating on the air was nelegected. When it was heating in the *i*th heating region (*i* = 1, 2, …, *n*. *n* = 25), the temperature in the *i*th heating region was set as *T*_flam_, and the temperatures in the other heating regions were set as *T*_room_ (i.e., the temperature of room). The heating source moved into next the heating region [i.e., the (*i* + 1)th heating region] after heating time *t*_flame_. Then, in the (*i* + 1)th heating region, the calculation/simulation mentioned in the *i*th heating region was repeated. The above procedures were repeated until the heating in the *n*th heating region was achived. The billet was heated by an oxygen-acetylene flame gun. The flame temperature of this particular gun is about 2000–3000 °C according to the study by Zhu et al [32]. The heat transfer in the FE model was an approximately ideal state. Thus, considering the heat loss, 2000 °C was adopted for the heating source. That is, the *T*_flame_ = 2000 °C was adopted in this study. The heating time in one heating region was 10 s, i.e., *t*_flame_ = 10 s. The temperature of the room/air was 20 °C.

The main form of heat exchange was forced-convection heat transfer during the heating process by the oxygen-acetylene flame gun. The physical properties of the AA5083 aluminum alloy, such as specific heat, thermal conductivity, and emissivity, are listed in Table 1. The convection heat transfer coefficient could be determined by Newton’s law of cooling:

(3) q=hΔT

where *q* is the heat flux density; *h* is the heat transfer coefficient; ΔT is the temperature difference between the transfer surface.

In order to facilitate the measurement, the heat transfer coefficient and the temperature could be described by dimensionless Nusselt number *Nu* [33]:

(4) $$Nu=\frac{hl}{\lambda }$$

where λ is the thermal conductivity of material (AA5083 aluminum alloy in this study); *l* is the characteristic length of transfer surface, which is surface area (*A*) of the heating source divided by the perimeter (*P*) of the heating source:

(5) $$l=\frac{A}{P}$$

Viskanta et al. [33] declared that the Nusselt number was about 100 for heating by flame spray gun. Considering the geometry of the heating source shown in Figure 6, the heat transfer coefficient was about 468,000 W/(m^2^·°C). Referring to relevant studies [31,34,35], the heat transfer coefficient between the heat source and the air was chosen as 40 W/(m^2^·°C). The parameters about heat in the local heating model are listed in Table 3.

### 3.4. FE Modeling of Warm Flanging

**Figure 7:**
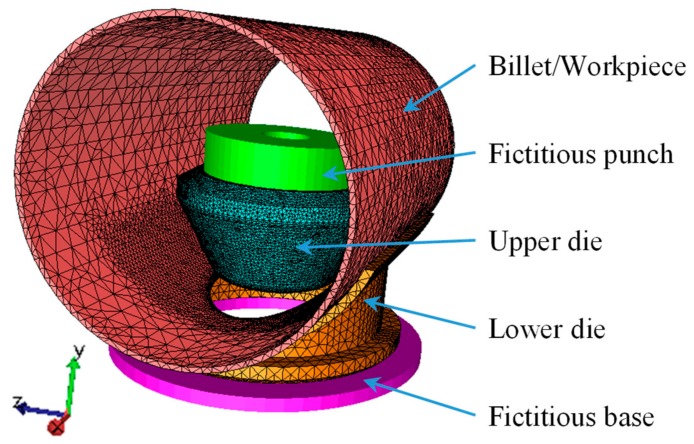
FE model of flanging process.

The material parameter, the geometric parameter, and the mesh generation for the billet/workpiece, the upper die, and the lower die are described in Section 3.1 and Section 3.2. The upper die and the lower die were elastic-plastic bodies in the FE model, thus a fictitious punch and a fictitious base were used to enforce constraints on the upper die and the lower die during FE modeling, respectively. The fictitious punch and the base did not make contact with the billet/workpiece, thus they were treated as a rigid body. Figure 7 illustrates the assembly relationship among the five parts and the meshes.

The temperature field after the second stage was the initial temperature field of the flanging process. There existed a heat exchange among the workpiece, the dies, and the air due to temperature differences. The heat transfer coefficient between the workpiece and the dies was 10,000 W/(m^2^·°C), and the heat transfer coefficient between the workpiece, the dies, and the air was 10 W/(m^2^·°C), as listed in Table 3.

A lubricant, such as castor oil, was applied to the forming area before heating. The Tresca friction model was adopted to describe the friction on the interface between the workpiece and the dies. According to the friction condition used in the numerical simulations for warm or hot forming of aluminum alloy [25,29,34,35] and considering the relationship [36] of friction condition between the Coulomb friction model and the Tresca friction model, the friction factor *m* = 0.2 was adopted in this study. The velocity of the punch was 0.5 mm/s, which pushed the upper die down. The stroke (*s*) of the punch was about 550 mm.

### 3.5. FE Modeling of Springback

**Figure 8:**
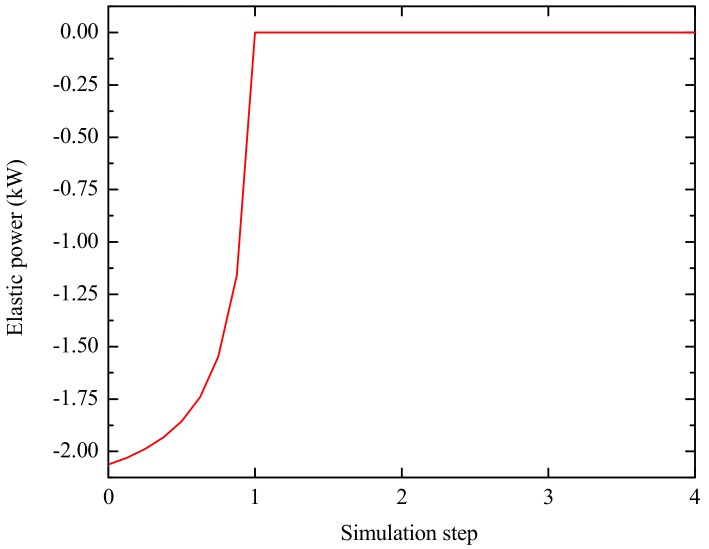
Elastic power during springback process.

After flanging, the dies were unloaded, and workpiece was on springback. The springback was done in a snap. During the fourth stage, the dies were removed, and the workpiece was elastic unloaded without a constraint condition. The release of elastic power was adopted to describe the springback process. The elastic energy accumulated in the flanging process released in one simulation step, as shown in Figure 8. The elastic unloading process was achieved in a snap, and thus it had no time, i.e., *t*_4_ = 0 s.

### 3.6. FE Modeling of Cooling

During the through warm flanging process of GIS shell, the cooling phenomenon appeared at two stages. One was the second stage after local heating. At this stage, the dies were transferred and installed, and the billet waited and cooled. The forced-convection heat transfer during the heating process had an influence on this stage. The other was the fifth stage after forming. At this stage, air cooling was adopted for the workpiece.

The temperature field after flame heating was the initial temperature field of the workpiece at the second stage, and the simulated flame condition was removed. The temperature field after flanging was the initial temperature field of the workpiece at the fifth stage, and the springback was achieved.

The heat transfer coefficients between workpiece and air at these two stages were different. The heat transfer coefficient between billet and air was about 10 to 40 W·m^−2^·K^−1^ according to the handbook, some reports, and the recommended value by commercial FE codes. Considering the influence of forced-convection heat transfer during heating, 40 W·m^−2^·K^−1^ was adopted at the waiting stage before the flanging process, and 10 W·m^−2^·K^−1^ was adopted during the flanging and the cooling processes. The influence of heating on the air was also nelegected. The material parameters, the heat parameters, and the process time are listed in Table 1 to Table 3.

## 4. Results and Discussion

### 4.1. Forming Defects and Its Control

#### 4.1.1. Typical Forming Defect

**Figure 9:**
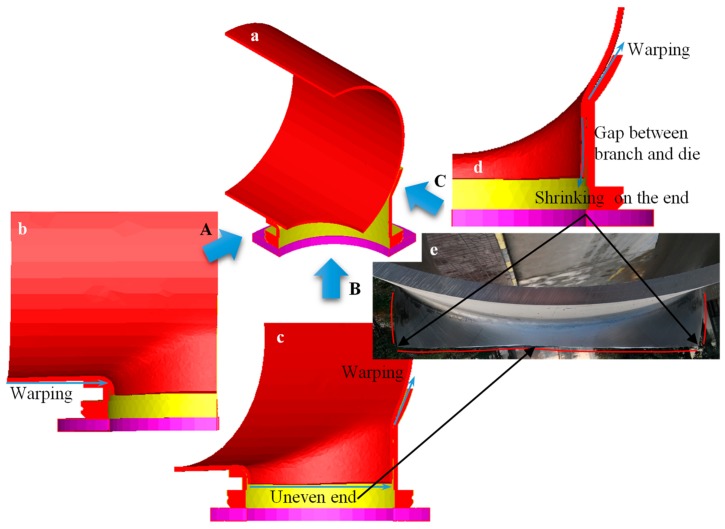
Representative defect in flanging with integral die: (**a**) quarter geometries of billet and dies; (**b**) view along A direction; (**c**) view along B direction; (**d**) view along C direction; (**e**) physical GIS shell.

The previous numerical study [25] indicated that there were three typical defects for the flanged branch by the whole mandrel (i.e., upper die): warping of the main pipe, as shown in Figure 9b, uneven end of the branch pipe, as shown in Figure 9c, and shrinking on the end of the branch pipe, as shown in Figure 9d. These were corroborated from the shapes of formed GIS shell parts. In this paper, when the forming defects were analyzed by combining the workpiece and the dies, the numerical results indicated that warping of the main pipe not only occurred along the axis (*x* direction) of the main pipe, as shown in Figure 9b, but it also occurred along the *y* direction, as shown in Figure 9c and d. The maximum of the former warpage appeared near the major axis (*x* axis) of the elliptical prefabricated hole, and the maximum of the latter warpage appeared near the minor axis (*z* axis) of the elliptical prefabricated hole. Generally, the maximum of shrinking on the end of the branch pipe appeared near the *z* axis. In addition, the sticking to the lower die for the branch pipe was not good due to the warpage at the root and the shrinking on the end of the branch pipe, because then the barrel was prone to occurrence in the middle of the branch. Because the constraint and the deformation for the major and the minor axes of the elliptical prefabricated hole were not coordinated, the roundness deviation of the branch pipe occurred after flanging forming.

The deformation resistance of the workpiece in the root area of the branch pipe was smaller than that of other areas of the main pipe. When the upper die moved downward (negative *y* direction), the vertical sidewall of the branch pipe had a corresponding downward pulling force. There was no blank holder device inside the workpiece, thus the contact between the root fillet area (The area is close to the vertical sidewall of lower die and even at the vertical sidewall.). of the branch and the lower die formed a fulcrum, and then the root of the branch was bended forward toward the positive *y* direction. There were two types of warping of the main pipe; the first type caused the diameter of the main pipe to be uneven along the axial direction (*x* direction), and the second type mainly concentrated on the corresponding area (around *z* axis) of the branch pipe.

**Figure 10:**
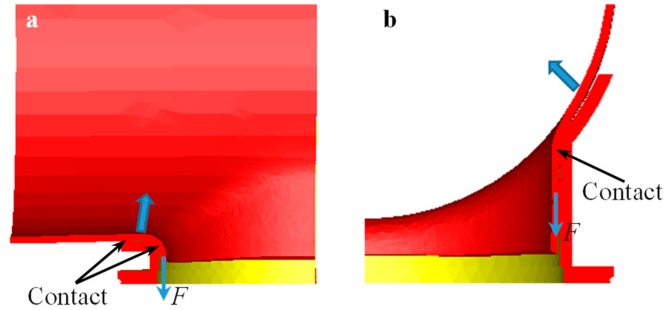
Mechanism of warping of the main pipe, the area around (**a**) the major axis and (**b**) the minor axis of the elliptical prefabricated hole.

The *x* axial supporting area of the lower die was small, and the *x* axial supporting length was short. During the flanging process, especially at the later stage, a valid contact appeared at the edge of the *x* axial support, thus the material between the transition fillet of the root of the branch and the edge of the *x* axial support of the lower die was haunched up to form the first type of warping phenomenon of the main pipe, as shown in Figure 10a. The die stress analysis showed that the maximum stress was concentrated on the edge of the *x* axial support of the lower die at the later stage of the flanging process. Similarly, the area around the minor axis of the elliptical prefabricated hole had a downward pulling force, and contact between the root fillet area of the branch and the lower die formed a fulcrum. Then, the main pipe contracted along the radial direction to form the second type of warping phenomenon of the main pipe, as shown in Figure 10b. The first type of warpage defect could be effectively controlled by increasing the length of the lower die in the *x* direction [25]. The second type of warpage defect could be controlled by optimizing the geometric parameters of the upper and the lower dies.

It is clear from Figure 9c that the height of the forming branch around the major axis of the prefabricated hole was slightly lower than that around the minor axis of the prefabricated hole. This was because the area around the minor axis (*z* axis) first contacted with the die and deformed during the flanging process. After the deformation was almost completed, the area around the major axis (*x* axis) of the prefabricated hole began to make contact with the die and deform. A small amount of material around the major axis of the prefabricated hole flowed to the area around the minor axis of the prefabricated hole at the initial stage of the flanging process, which could be evidenced by the velocity field analysis. The differences in times when severe plastic deformation began in the area around the major and the minor axes of the elliptical prefabricated hole could be reduced by reasonable design of the prefabricated hole. Then, this defect, i.e., uneven end of the branch pipe, could be reduced and eliminated. However, the dimension of the elliptical prefabricated hole should have considered the geometrical parameters of the forming branch, such as height and diameter.

The non-sticking, the shrinking on the end, and the barrel in the middle of the branch pipe were problems mainly caused by inhomogeneous circumferential deformation of the branch. These defects could be improved by adjusting the gap between the upper die and the lower die. However, it was difficult to adjust the flanging used in the integral upper die. It is a great challenge to completely eliminate these defects by optimization of the flanging process.

#### 4.1.2. Comparison between Predicted Results and Experimental Results

**Figure 11:**
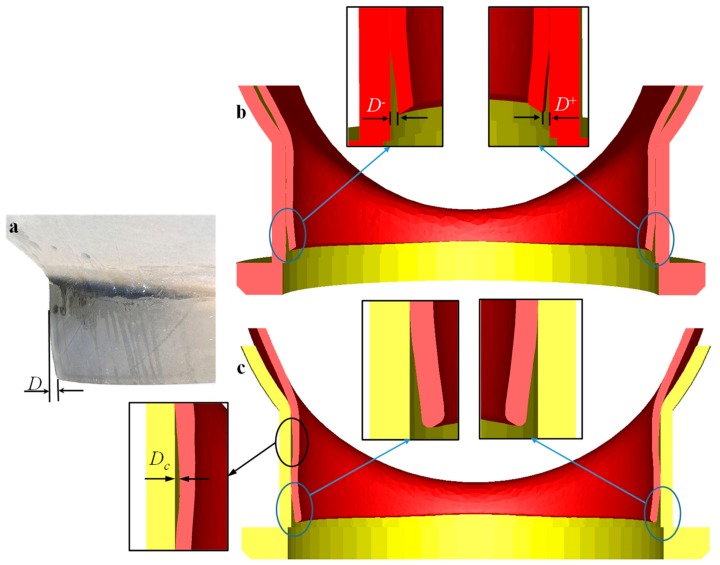
Shrinking on the end of branch pipe: (**a**) experiment; (**b**) finite element method (FEM) result by the modeling method in this study; (**c**) FEM result by the modeling method in the study [25].

Shrinking on the end of the branch pipe was a typical macroscopic defect, as shown in Figure 11a. The FE model developed in Section 3 and developed in the study [25] both predicted this macroscopic defect, as shown in Figure 11b,c. The degree of shrinking on the end of the branch pipe could be represented by the distance *D* from the end of the branch to the sidewall of the lower die (or the maximum outer diameter of the branch), as shown in Figure 11a,b. Figure 11 illustrates the comparison of shrinking between experimental results and FEM results.

Generally, the maximum of shrinking on the end of the branch pipe appeared near the *z* axis. That is to say, the maximum value of the index *D* generally appeared near the *z* axis. Although the force field conditions and the geometric conditions of the area near the *z* axis were consistent, the initial temperature fields were different, thus *D*^+^ and *D*^−^ were not consistent at the end, as shown in Figure 11b. The FE model developed in the study [25] could not predict the difference between them. In the following sections with the quantitative discussion of the effect of parameters on index *D*, the value of index *D* was calculated by Equation (6).

(6) $$D=\frac{D^{+}+D^{-}}{2}$$

**Figure 12:**
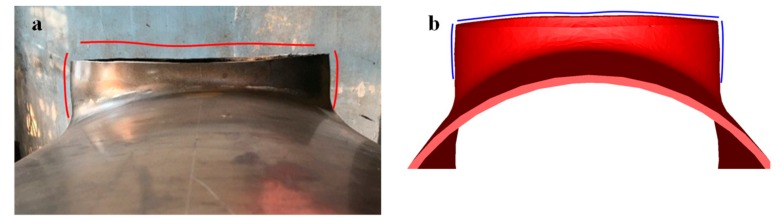
Shape of branch: (**a**) experiment; (**b**) FEM.

The FE model developed in Section 3 could also predict the uneven end of the branch pipe and the barrel in the middle of the branch pipe, as shown in Figure 12. These accurate predictions for macroscopic defects indicated that the established FE model could describe the deformation behavior of the warm drawing process.

**Figure 13:**
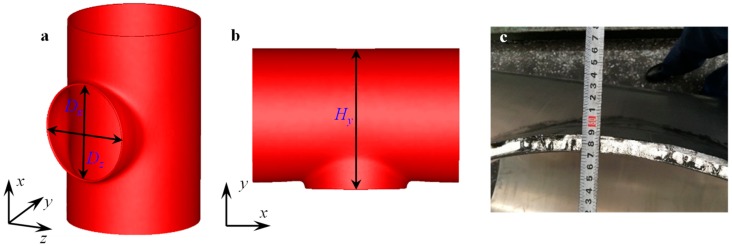
Representation and measurement of geometry of branch pipe: (**a**) diameter; (**b**) height; (**c**) actual measurement.

The representation indexes *D_x_* and *D_y_* for branch diameter were determined according to the roundness deviation of the branch and the shape of the prefabricated hole mentioned above, as shown in Figure 13a. The height of the branch was represented by *H_y_* along the y axis, as shown in Figure 13b. The detailed measurements of the simulation results and the experimental results are listed in Table 4. There were obvious dimensional changes before and after springback, and there were only slight dimensional changes during the cooling process. Compared with the experimental results, the maximum relative error after cooling was about 0.3%. The established FE model in Section 3 could accurately describe the macroscopic deformation characteristics of the through process of GIS shell warm flanging.

#### 4.1.3. Control of Forming Defect

The gap between the upper die and the lower die was directly determined by the radius *R*_u_ (shown in Figure 4) of the sizing section of the upper die. The gap played a role in metal flow during the flanging process and had more or less influence on the forming defects mentioned above. It had the greatest influence on the index *D* (shown in Figure 11a,b) and the geometric parameters of the branch pipe. The previous study [25] paid attention to the degree of non-sticking of the branch pipe, which was represented by the distance *D_c_* (shown in Figure 11c) between the outer wall of the branch pipe and the inner wall of the lower die. The index *D_c_* decreased as the radius *R*_u_ increased.

In general, the radius of the sizing section of the upper die should satisfy Equation (7).

(7) $$R_{u}\leq R_{l}-t$$

where *R_l_* is the inner diameter of round hole of lower die; *t* is the thickness of the formed workpiece, and the initial thickness of billet is often used.

The roundness deviation of the branch pipe occurred after flanging due to uncoordinated constraint and the deformation for the long and the short axes of the elliptical prefabricated hole. The index Δ*D_xz_* (Equation (8)) could be used to describe the degree of non-circularity of the branch pipe after the drawing and flanging forming.

(8) $$\Delta D_{xz}=D_{x}-D_{z}$$

**Figure 14:**
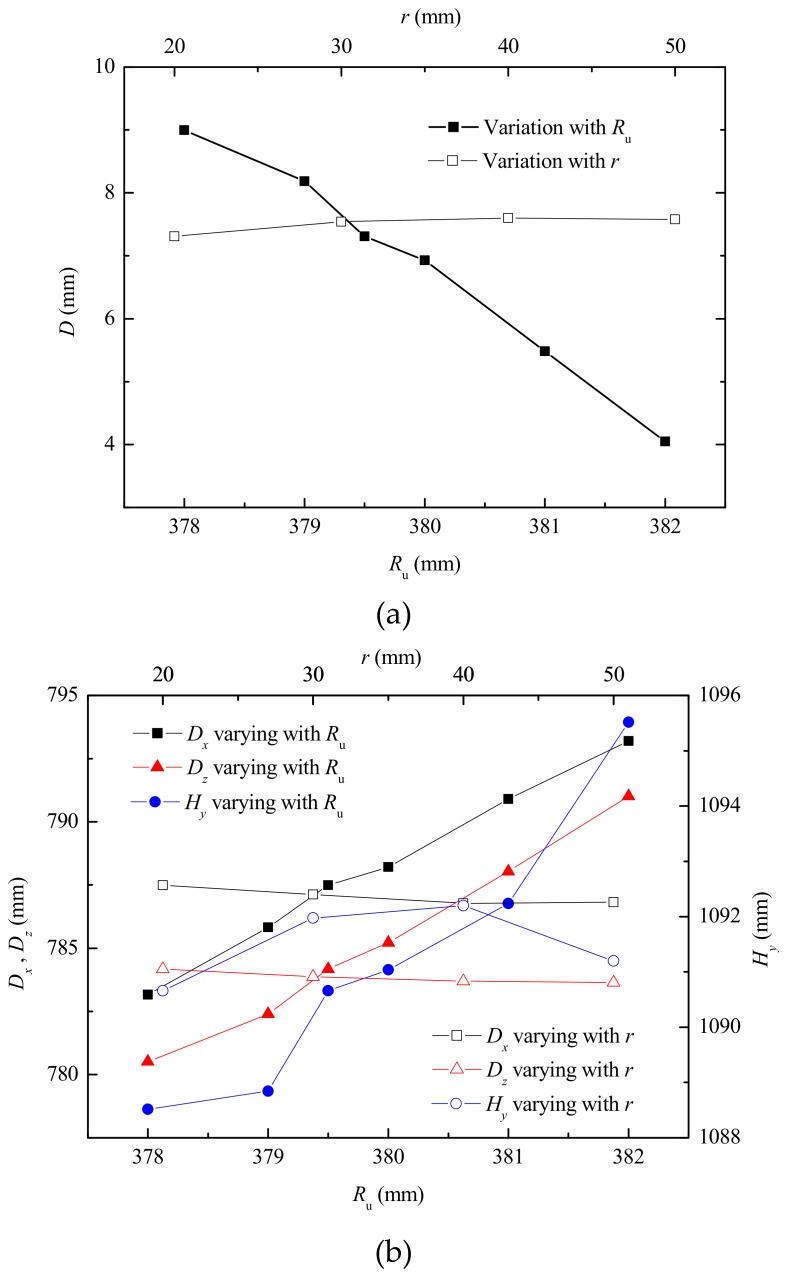
Influence of geometry of upper die (**a**) on index *D* and (**b**) on size of branch.

According to Equation (7), the maximum value of *R*_u_ was 382 mm. Based on this, several sets of *R*_u_ were selected to study the effect on the forming performance of the flanging. As *R*_u_ increased, the index *D* was significantly reduced, as shown in Figure 14a. Ben et al. [25] used the index *D*_C_ to describe the degree of non-sticking, which also reflected the degree of the barrel in the middle of the branch. As *R*_u_ increased, the indicator *D*_C_ was also reduced [25]. Increasing the transition radius *r* between the sizing section and the forming section of the upper die could improve the uniformity of deformation. However, there was no significant contribution to the index *D*, as shown in Figure 14a.

With the increase of the radius *R*_u_ of the upper die, the gap between the upper die and the lower die was reduced, and the material fully contacted the die. Increasing the *R*_u_, the diameter of the final formed branch pipe almost linearly increased, and the height of the branch also increased, as shown in Figure 14b. However, the degree of increase in *D_x_* and *D_z_* was almost the same. In other words, the increase of *R*_u_ did not change the roundness deviation of the branch pipe after flanging. Similarly, it is clear from Figure 14b that the transition radius *r* between the upper sizing section and the forming section had little effect on the size of the branch pipe.

**Figure 15:**
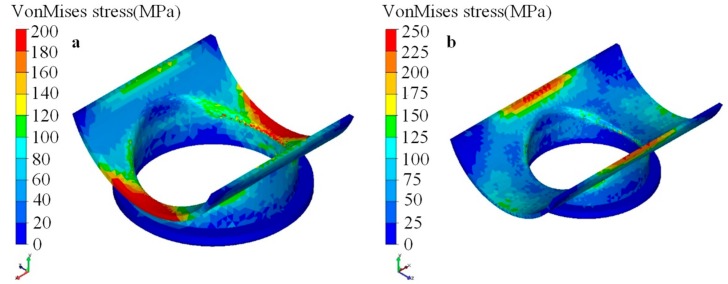
Distribution of stress for lower die with different length (stroke was 430 mm): (**a**) short *x* length; (**b**) long *x* length.

**Figure 16:**
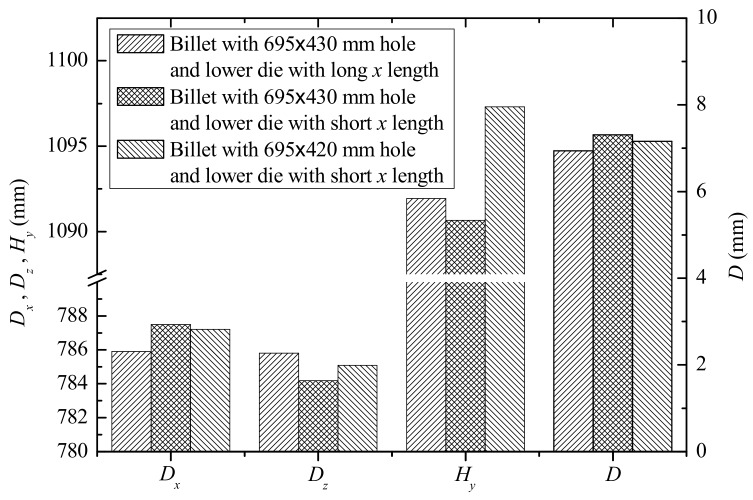
Size of branch flanged by different elliptic pre-holes and geometries of lower die.

By increasing the *x* length (i.e., the length along the axial direction of the main pipe) of the lower die, the first warpage could be significantly reduced [25]. The stress distribution of the lower die changed greatly with increasing *x* length. The stress distribution of the contact zone of the lower die along the *x* direction was more uniform, and the maximum stress zone was also transferred to the corresponding zone around the *y* axis, as shown in Figure 15. Figure 15b indicates that the contact (which resulted in second warpage) between the die and the workpiece was enhanced, and this may have reduced the degree of the second warpage. Increasing the *x* length of the lower die also significantly improved the roundness deviation of the branch, and Δ*D_xz_* was less than about 0.2 mm, as shown in Figure 16. However, the influence on the branch’s height and shrinking on the end was little.

However, increasing the *x* length of the lower die not only increased the cost of die processing and installation and extended the time, but it also limited the structure of the adjacent region of the formed branch pipe. According to the above analysis, *D_z_* was less than *D_x_* after the warm flanging process, thus material around *D_z_* could be increased. In other words, the minor axis dimension of the elliptical prefabricated hole was reduced. Figure 16 illustrates the size of the branch pipes flanging by different prefabricated holes. The results indicated that the roundness on end of the formed branch pipe was improved, and it was closer to the target size of the branch pipe. The height of the branch was also increased due to more metal around the minor axis of the prefabricated hole. However, it had no significant improvement on the shrinking on the end.

There were five kinds of forming defects—warping of the main pipe around the root of the branch, the barrel in the middle of the branch, shrinking on the end of the branch, uneven end of the branch, and roundness deviation of the branch—for the warm flanging process of large-size aluminum alloy GIS shells, where warping could be classed into two types, namely warping around the axial (*x* axis) and the radial (*y* axis) directions of the main pipe. The shrinking could be significantly improved by increasing the diameter of the sizing section of the upper die. The warping and the roundness deviation could be significantly improved by increasing the *x* length (i.e., the length along the axial direction of the main pipe) of the lower die. The roundness deviation also could be improved by size optimization of the prefabricated hole.

### 4.2. Evolution of Temperature Field for Workpiece

**Figure 17:**
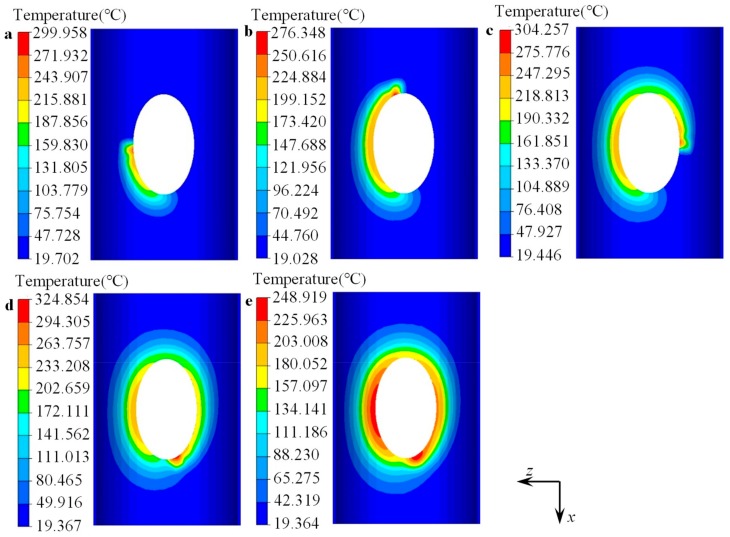
Distribution of temperature at chosen stages before flanging process: local heating (**a**) 25%, (**b**) 50%, (**c**) 75%, (**d**) 100%; (**e**) after waiting.

The heating process for the forming zone of the cylindrical billet was simulated by the FE model developed in Section 3.2, and the uneven distribution of the temperature along the elliptical prefabricated hole could be predicted. The FE model could better reflect the actual heating process. Five typical stages were selected to reflect the temperature change in the forming zone of the workpiece before flanging, as shown in Figure 17. Figure 17 displays that the heated region increased as the heating source moved. During the whole heating process, the maximum temperature of the cylindrical billet was about 300 °C. After the heating process, the temperature in most areas around the elliptical prefabricated hole was about 200 °C, as shown in Figure 17d.

During the waiting stage for die installation (i.e., the second stage), the temperature of the cylindrical billet decreased slightly. The maximum temperature was less than 250 °C, and the temperature of the peripheral area of the elliptical prefabricated hole was above 150 °C, as shown in Figure 17e, which met the temperature requirement for the flanging process. After local heating of the workpiece, the temperature gradually decreased to the ambient temperature from the edge of the prefabricated holes to the farside. It only had a certain temperature in the peripheral region of the prefabricated hole, and the temperature distribution along the circumferential direction of the prefabricated hole was also not uniform.

**Figure 18:**
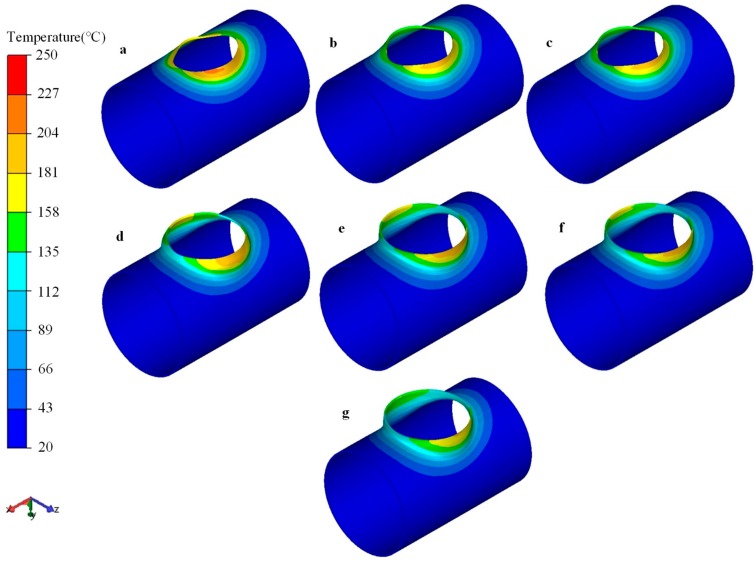
Distribution of temperature at chosen stages during flanging process, strokes of upper die were (**a**) 100 mm, (**b**) 200 mm, (**c**) 315 mm, (**d**) 350 mm, (**e**) 430 mm, (**f**) 450 mm, (**g**) 500 mm.

Figure 18 illustrates the temperature evolution of the workpiece during the flanging process. The temperature of the die was room temperature, which was much lower than the temperature of the contact zone. The temperature of the workpiece dropped significantly under the combined action of chilling the die surface and natural cooling. However, severe plastic deformation generated heat, and it compensated for some temperature loss. The area around the minor axis (*z* axis) of the elliptic prefabricated hole first contacted with the die and deformed during the flanging process. Therefore, the dropping degree of temperature in the deformed region around the minor axis (*z* axis) of the prefabricated hole was smaller than that around the major axis (*x* axis) of the prefabricated hole. During the flanging process, the temperature of most deformation zones could be maintained at about 150 °C, and the temperature in the area around the major axis (*x* axial) of the prefabricated holes could also be guaranteed above 110 °C, as shown in Figure 18.

**Figure 19:**
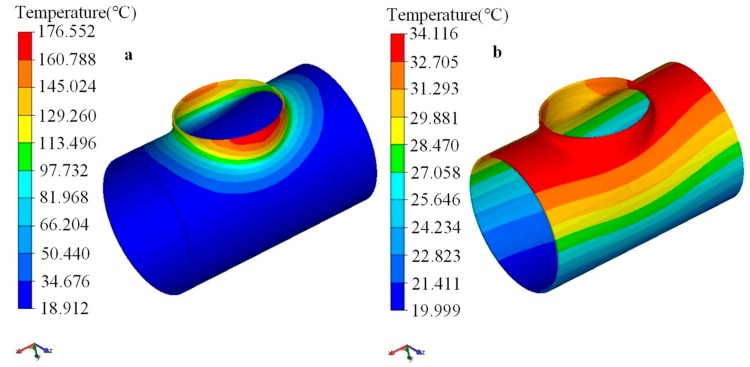
Distribution of temperature during cooling process: (**a**) after flanging, (**b**) 2000 s cooldown.

The maximum temperature of the workpiece was about 170 °C after the flanging process, and the temperature of the formed branch pipe was generally greater than 100 °C, as shown in Figure 19a. During the cooling period, the temperature dropped sharply in 300 s. After cooling for 2000 s, the maximum temperature of the workpiece was about 30 °C, as shown in Figure 19b, which indicated that the cooling process was finished.

**Figure 20:**
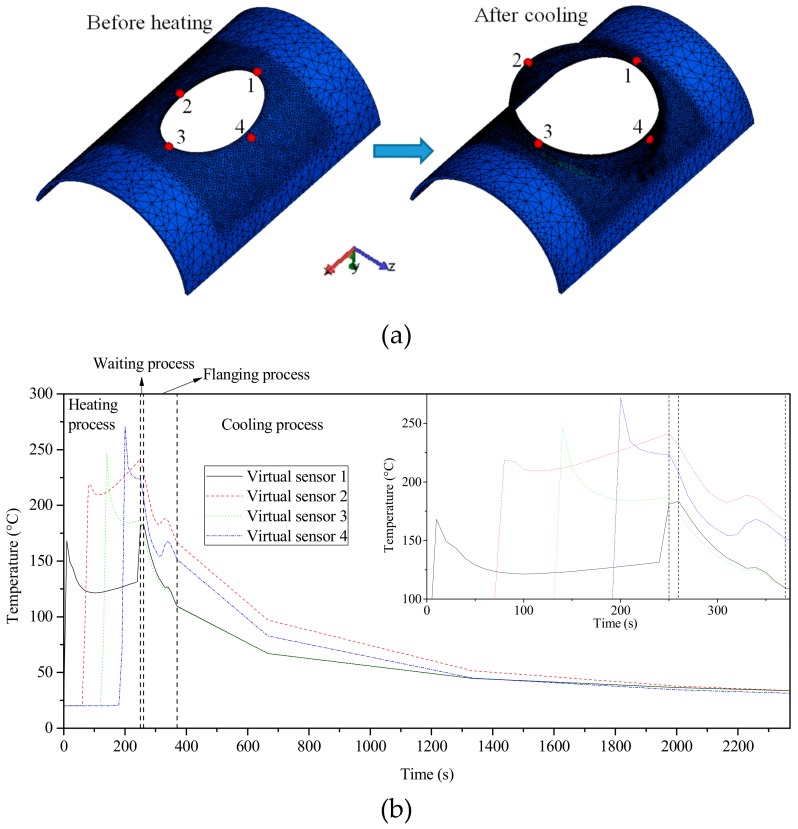
Temperature of virtual sensor during whole process: (**a**) position of virtual sensor; (**b**) temperature–time curve.

In order to understand the evolution of the temperature in the deformation region during the through process more clearly, four points were selected in the forming area to arrange the virtual sensors according to the heating sequence. The virtual sensors moved with metal flow during the deformation process, as shown in Figure 20a. Figure 20b illustrates the temperature variations recorded by the four virtual sensors during the through process.

Figure 20b shows that when the heating source moved closer to the virtual sensor, the measured temperature rose linearly. When the heating source moved to the next area, the temperature recorded by the virtual sensor slowly decreased. After the measured temperature decreased for a while, the temperature rose slowly because the front area of the virtual sensor was continuously heated. At the end of the local heating process, the heating source re-approached to the initial heating area, and thus the temperature of virtual sensor 1 rose sharply near the end of heating process.

During the waiting and cooling stage (i.e., the second stage), the temperature in each zone generally dropped because the flame gun stopped working. However, the temperature rising effect still acted on virtual sensor 1, and its temperature did not drop; in fact, it even rose slightly (2.7 °C).

During the whole flanging process, the temperature showed a downward trend. However, due to the heat generated by plastic deformation, the temperature of the virtual sensors slightly rose some time during the flanging process, especially at the position of the virtual sensors 2 and 4.

During the whole cooling process, the temperature of the workpiece dropped sharply. The temperature dropped to about 80 °C after cooling for 300 s. Then, the cooling rate reduced, and the temperature dropped to about 45 °C after cooling for 1000 s. After that, the air cooling effect was poor, and the temperature dropped very slowly. After cooling for 2000 s, the final temperature of the workpiece was reduced to about 30 °C, which could be regarded as the end of the cooling process.

### 4.3. Deformation Characteristics of Flanging Process

#### 4.3.1. Load and Contact

**Figure 21:**
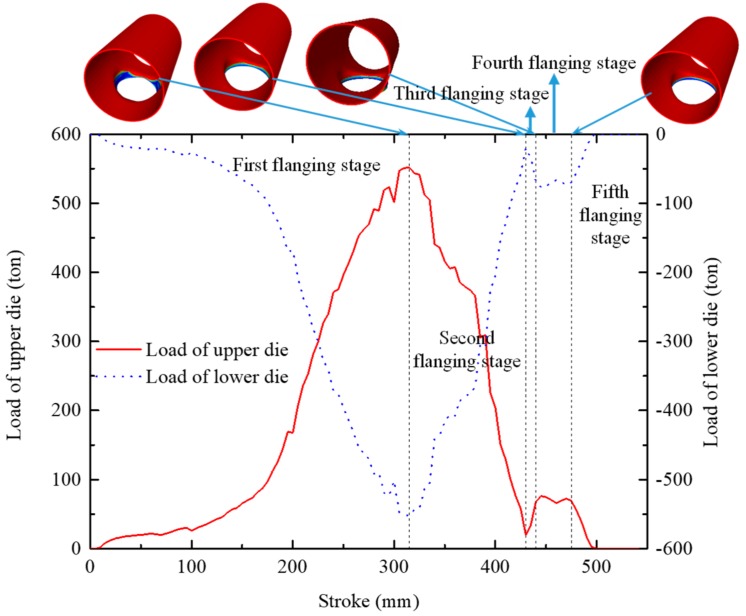
Load of upper die during flanging process.

The load along the loading direction (*y* axis) of the die was the maximum forming load among loads along three directions, which were important parameters for selecting or designing flanging equipment. The loads discussed in this section are the forces along the loading/flanging direction. At the same forming time, the loads of the upper die and the lower die were almost equal in magnitude and opposite in direction, as shown in Figure 21.

As the stroke (*s*) of the upper die increased, the forming load gradually increased. When the stroke reached 315 mm, the forming force reached a maximum at this time, which was about 550 T. After that, as the stroke of the upper die increased, the forming force gradually decreased. When the stroke reached 430 mm, the forming force began to rise slightly. After the force increased to a certain value, the forming force remained unchanged for a certain time and then fell to zero until the end of the flanging process.

According to the variation of the forming force, the flanging forming process could be divided into five forming stages. At the first stage of flanging, the forming force rose sharply until it reached the maximum; at the second stage of flanging, the forming force dropped sharply; at the third stage of flanging, the forming force rose slightly; at the fourth stage of flanging, the forming force was almost a constant (about 70 ton); at the fifth stage, the forming force dropped again until it dropped to zero. The varying characteristics of the forming force were closely related to the contact state between the workpiece and the die.

**Figure 22:**
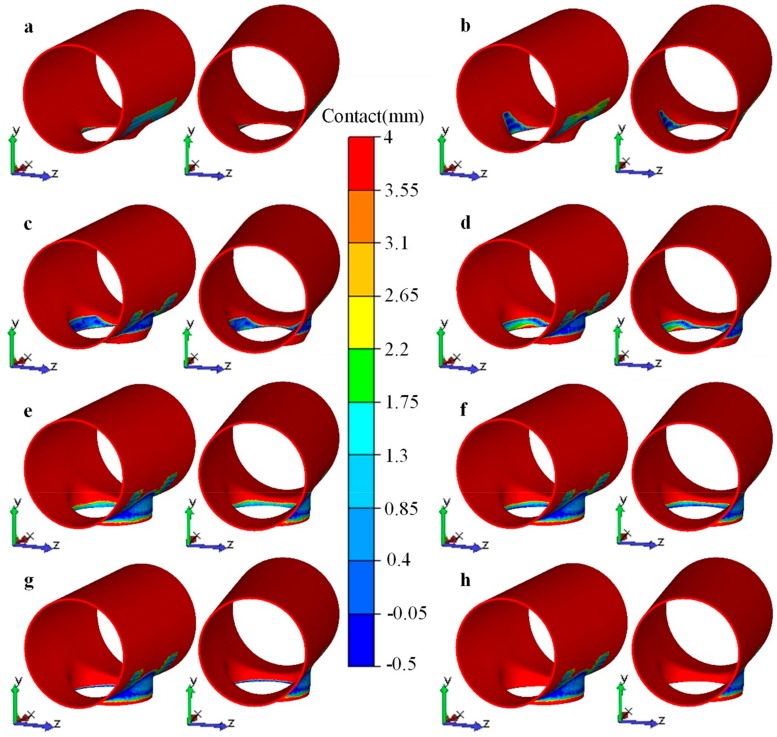
Contact between workpiece and upper/lower dies at chosen stages during flanging process, strokes of upper die were (**a**) 100 mm, (**b**) 200 mm, (**c**) 315 mm, (**d**) 350 mm, (**e**) 430 mm, (**f**) 440 mm, (**g**) 475 mm, (**h**) 500 mm.

Figure 22 illustrates the contact state between the workpiece and th edie during the flanging process. At the first stage of flanging, when the flanging forming started, the first contact with the upper die was the material around the minor axis of the prefabricated hole, and the material began to bend and deform, as shown in Figure 22a. As the stroke of the upper die increased, the area contacting the upper die around the minor axis (*z* axis) gradually increased, and the material participating in the bending deformation gradually increased, as shown in Figure 22b, thus the forming force rapidly rose. When the stroke reached 315 mm, the material around the major axis (*x* axis) of the prefabricated hole also began to make contact with the upper die, as shown in Figure 22c, and the material began to bend and deform, thus the forming force reached a maximum at this time.

However, when the stroke reached 315 mm, the deformation of the branch pipe around the minor axis of the prefabricated hole was substantially completed. At the second stage of flanging, the process was mainly used for sizing and forming a vertical side. The material at the end of the branch pipe (the area near the minor axis of the prefabricated hole) may have disengaged from the upper die, as shown in Figure 22d. Although the new contact area around the major axis of the prefabricated hole steadily extended, the disengaged contact area around the minor axis of the prefabricated hole was even larger. At the end of the second stage (i.e., stroke near 430 mm), the material in the end region of the branch contacted with the vertical side of the upper die again, as shown in Figure 22e, but the contact area was reduced. Therefore, the forming force continuously dropped at the second stage of flanging.

**Figure 23:**
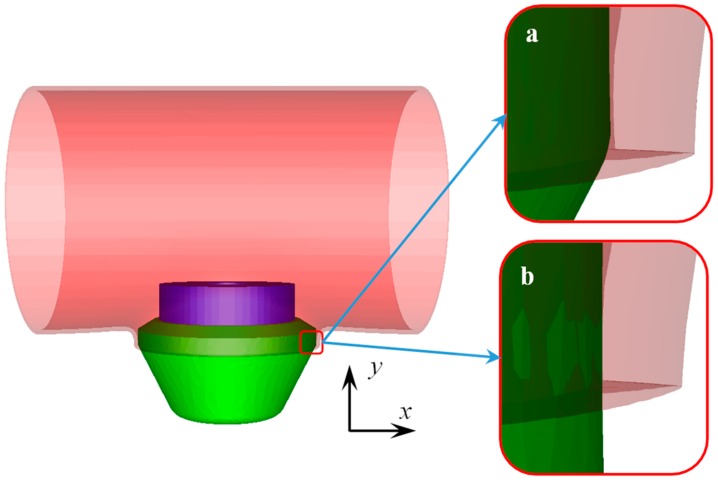
Contact of the end of the branch at the third and the fourth flanging stages, strokes of upper die were (**a**) 430 mm, (**b**) 450 mm.

At the third stage of flanging, only the material around the end of the branch contacted with the upper die, and the material of other portions were formed. The material around the end of the branch began to contact with the sizing section of the upper die, as shown in Figure 23a, and the contact was gradually strengthened and stabilized, as shown in Figure 23b. Therefore, it showed that the force rose slightly and stabilized at a certain value.

Then, the deformation of the branch was done, thus the workpiece gradually disengaged from the upper die until there was no contact, as shown in Figure 22h. Therefore, the forming force gradually dropped to zero at the fifth stage of flanging.

#### 4.3.2. Evolution of Stress and Strain Fields

**Figure 24:**
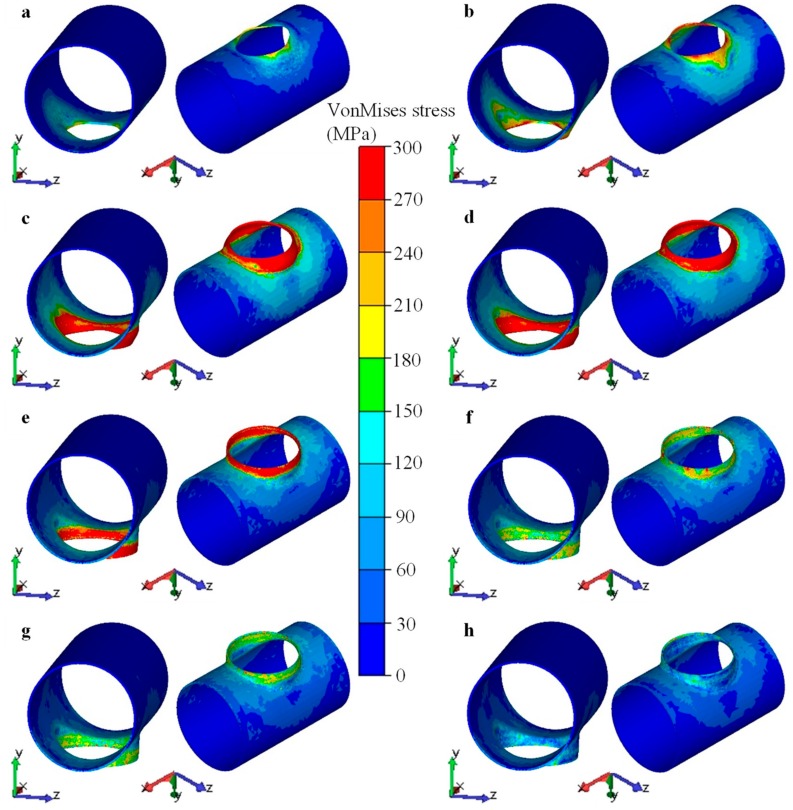
Distribution of stress for workpiece at chosen stages during flanging process, strokes of upper die were (**a**) 100 mm, (**b**) 200 mm, (**c**) 315 mm, (**d**) 350 mm, (**e**) 430 mm, (**f**) 440 mm, (**g**) 475 mm, (**h**) 500 mm.

Figure 24 illustrates the distributions of stress for the workpiece at chosen stages of the flanging process. The region around the minor axis (*z* axis) of the elliptical prefabricated hole contacted with the upper die at first, thus the stress in this region gradually increased and expanded along the circumferential direction of the prefabricated hole. The evolution of the stress field was similar to the evolution of the contact area. When the stroke of the upper die reached 315 mm, the area of large stress (>270–300 MPa) reached the maximum, and the forming force also reached the maximum. Then, the area of large stress gradually reduced, and the forming force also dropped. When the stroke reached 430 mm, most of the deformation for the the branch was completed. Only the end of the branch—especially the area around the major axis (*x* axis) of the elliptical prefabricated hole—had a few portions that needed a final sizing and flanging. Therefore, there were a few large-stress regions at the later stage. The maximum stress was generally less than 240 MPa, and the stress in most regions was less than 200 MPa. 

**Figure 25:**
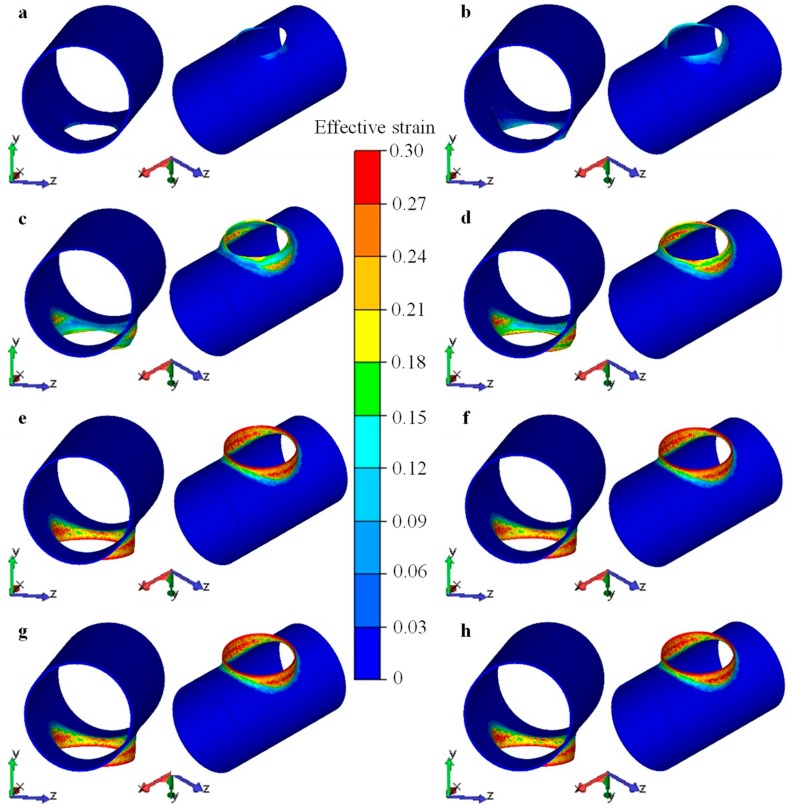
Distribution of strain for workpiece at chosen stages during flanging process, strokes of upper die were (**a**) 100 mm, (**b**) 200 mm, (**c**) 315 mm, (**d**) 350 mm, (**e**) 430 mm, (**f**) 440 mm, (**g**) 475 mm, (**h**) 500 mm.

Figure 25 illustrates distributions of strain for the workpiece at chosen stages of the flanging process. The distribution and the evolution of the strain field for the workpiece during the flanging process were similar to those of the stress field. However, strain is a cumulative value, thus its value was continuously increasing. The metal yielded in the region around the minor axis (*z* axis) of the elliptical prefabricated hole at first. Then, the plastic deformation zone expanded along the circumference and the radial direction of the elliptical prefabricated hole. The plastic deformation zone was concentrated on the branch region, and there was a small transition zone adjacent to the main pipe, and the other zones did not yield. When the stroke of the upper die reached 430 mm, the deformation was basically completed. After that, only a small position near the end of the branch—especially the area around the major axis (*x* axis) of the elliptical prefabricated hole—had a plastic deformation. Therefore, the distributions of strain field almost remained unchanged, as shown in Figure 25e,h.

### 4.4. Analysis of Springback

**Figure 26:**
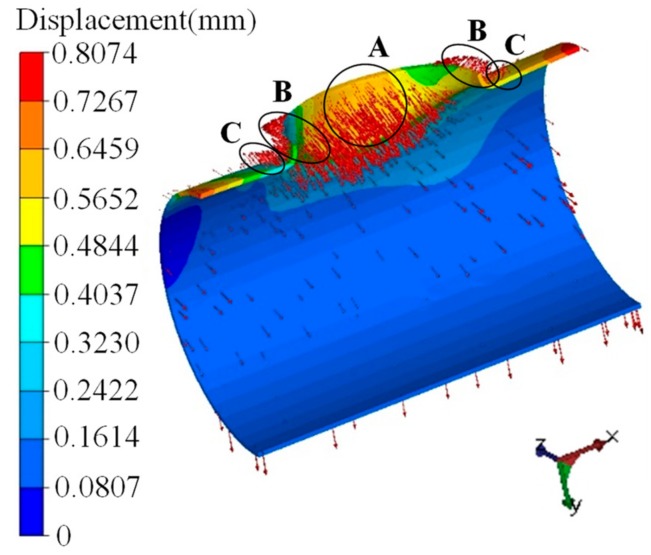
Displacement and velocity fields for springback.

Table 4 shows that the dimensional changes during the cooling process were much smaller than the dimensional changes during the elastic unloading. Therefore, this section focuses on the springback caused by the elastic unloading. During the process of the elastic unloading, the accumulated elastic energy of the flanging process was released in one simulation step, as shown in Figure 8. Figure 26 illustrates the displacement and the velocity fields after springback.

The springback mainly occurred near the branch. For the branch pipe area, the direction of the springback in region A around the minor axis (*z* axis) of the elliptical prefabricated hole was opposite to that in region B around the major axis (*x* axis) of the prefabricated hole. The springback of region A was towards the inside of the branch, while the springback of region B was towards the outside of the branch. From the *z* axis to the *x* axis, the springback direction gradually turned from inward to outward. The area (such as region C) of the main pipe near the *x* axis rebounded to the outside of the cylinder tube, and this displacement strengthened the forming defects, such as the warping of the main pipe.

The region A contracted along the radial direction of the branch, and then the diameter of the branch reduced. The region B expanded along the radial direction of the branch, and then the diameter of the branch increased. The changes of *D_x_* and *D_y_* before and after the springback listed in Table 4 were consistent with this trend. The changing trend for the diameter of the branch during the cooling process was similar to this. The region A shrunk along the axial direction (*y* axis) of the branch, and then the height of the branch reduced. The change of *H_y_* before and after the springback listed in Table 4 coincided with this trend. The changing trend for the height of the branch during the cooling process was similar to this. The region B expanded along the axial direction (*y* axis) of the branch, and then the height of the branch increased. Therefore, the degree of the uneven end of the branch pipe was reduced, and the previous study [25] also obtained similar conclusions for the height of the branch.

**Figure 27:**
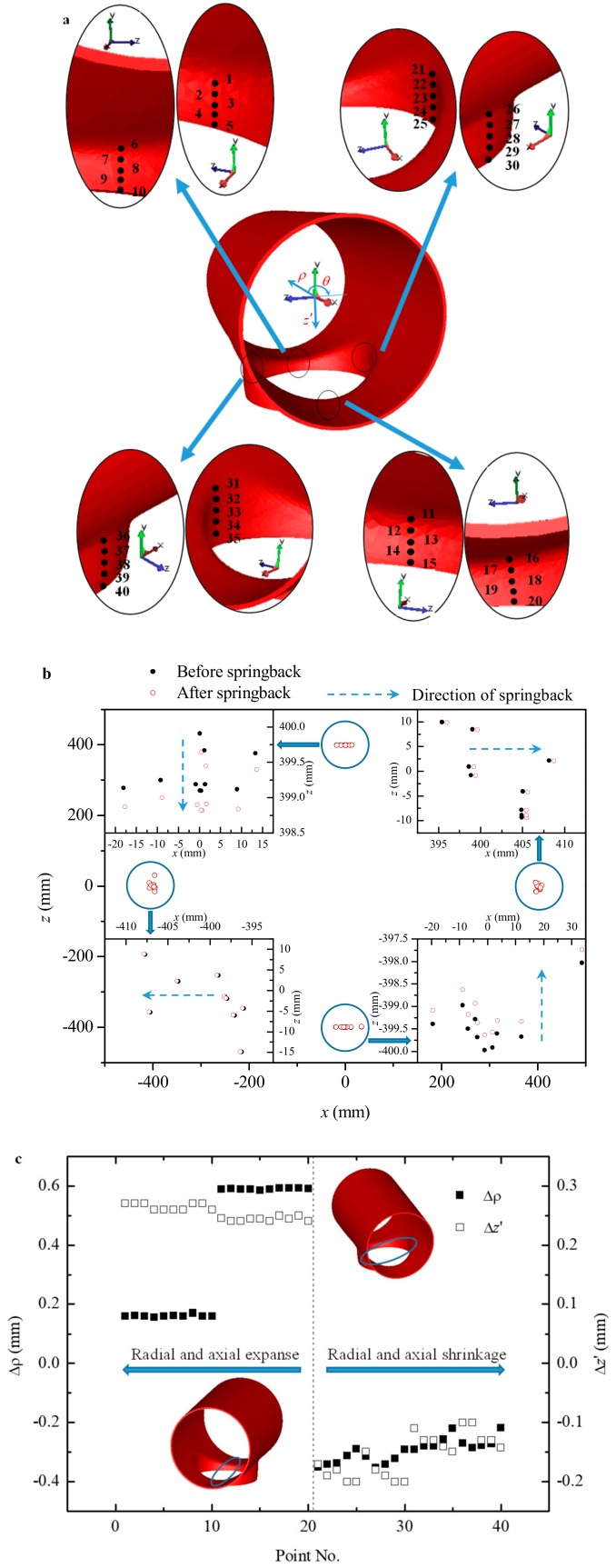
Springback of chosen points: (**a**) positions of points; (**b**) *xz* projection of chosen points; (**c**) quantitative calculation of springback.

According to the above analysis, springback of the branch in the region around the *x* and the *z* axes was notable, and characteristic springback in the region around the *x* axis was different from that in the region around the *z* axis. Thus, five points were chosen from inside and outside of the branch near the *x* and the *z* axes to quantitatively analyze the springback, where a total of 40 points were chosen, as shown in Figure 27a. In order to analyze the springback of the branch pipe, a local cylindrical-coordinate system $O\rho \theta z^{\prime }$ for the branch was established, as shown in Figure 27a. The local coordinates could be expressed by world coordinates as follows:

(9) $$\rho =\sqrt{x^{2}+z^{2}}$$

(10) $$z^{\prime }=-y$$

Figure 27b illustrates the world coordinates of the chosen points before and after springback. It also indicates that the branch pipe expanded along the *x* axis and shrunk along the *z* axis. The world coordinate was $P_{i}$($x_{i}$, $y_{i}$, $z_{i}$) before springback and was $P_{i}^{s}$($x_{i}^{s}$, $y_{i}^{s}$, $z_{i}^{s}$) after springback. The variation (Δρ) of the radial direction and the variation ($\Delta z^{\prime }$) of the axial direction for the branch after springback could be calculated by Equations (11) and (12), respectively. If the value of variation was negative, then the size shrunk along the radial (ρ) or the axial ($z^{\prime }$) direction; if the value of variation was positive, then the size expanded along the radial (ρ) or the axial ($z^{\prime }$) direction.

(11) $$\Delta \rho =\rho_{i}^{s}-\rho_{i}=\sqrt{{\left(x_{i}^{s}\right)}^{2}+{\left(z_{i}^{s}\right)}^{2}}-\sqrt{x_{i}^{2}+z_{i}^{2}}$$

(12) $$\Delta z^{\prime }={\left(z^{\prime }\right)}_{i}^{s}-{\left(z^{\prime }\right)}_{i}=-\left(y_{i}^{s}-y_{i}\right)$$

where *i* = 1, 2, …, 40.

Figure 27c illustrates variation along the radial or the axial direction of the branch after springback. The value of variation was positive for the points taken from the region near the major axis (*x* axis) of the elliptical prefabricated hole. It indicated that the branch in this region expanded along ρ, and the height increased along $z^{\prime }$. However, the value of variation was negative for the points taken from the region near the minor axis (*z* axis) of the elliptical prefabricated hole. It indicated that the branch in this region shrunk along ρ, and the height reduced along $z^{\prime }$. The roundness deviation of the branch increased after springback, and the degree of the uneven end of the branch reduced after springback.

## 5. Conclusions

- Based on FE soft FORGE code, a 3D-FE model for the heating-waiting-flanging-unloaded-cooling through-process was developed. The model was validated by comparison with geometric shapes and forming defects obtained from the experiment. The developed FE model could describe the inhomogeneous temperature field, the deformation behavior, and the springback along circumferential, radial, and axial directions of the formed branch. The finite element analysis of the warm flanging process was carried out by the developed 3D-FE model.
- Only the peripheral region of the prefabricated hole was heated, and the temperature along the radial and the circumferential directions of the elliptical prefabricated hole presented a notable inhomogeneous state. During the flanging process, the area around the minor axis (*z* axis) of the elliptical prefabricated hole contacted the die and deformed at first, thus the dropping degree of temperature in the region around the minor axis (*z* axis) was lower than that around the major axis (*x* axis) of the prefabricated hole. The deformed zone could maintain a suitable temperature for deformation during warm flanging.

## Figures and Tables

**Table 1:** Process and times at each stage.

	Process	Time
First stage	Local heating	*t*_1_ = 250 s
Second stage	Cooling	*t*_2_ = 10 s
Third stage	Deformation and heat transfer	*t*_3_ = 110 s
Fourth stage	Springback	*t*_4_ = 0 s
Fifth stage	Cooling	*t*_5_ = 2000 s

**Table 2:** Elastic and physical properties of materials.

Material	AA5083	H13
Elasticity modulus *E* (GPa)	73	210
Specific heat (J⋅kg^−1^⋅K^−1^)	1230	778
Thermal conductivity (W⋅m^−1^⋅K^−1^)	117	35.3
Thermal expansion coefficient (10^−6^⋅K^−1^)	23.75	10.9
Emissivity	0.05	0.88

**Table 3:** Parameters about heat in the simulation.

Process (stage)	Parameter	Value
Local heating (1st stage)	Temperature of heating source *T*_flame_ (°C)	2000
	Heating time of heating source *t*_flame_ (s)	10
	Heat transfer coefficient between billet and heat source (W⋅m^−2^⋅K^−1^)	468,000
	Heat transfer coefficient between heat source and air (W⋅m^−2^⋅K^−1^)	40
Heating and waiting (1st and 2nd stages)	Heat transfer coefficient between billet and air (W⋅m^−2^⋅K^−1^)	40
Flanging (3rd stage)	Heat transfer coefficient between workpiece and dies (W⋅m^−2^⋅K^−1^)	10,000
	Heat transfer coefficient between die and air (W⋅m^−2^⋅K^−1^)	10
	Initial temperature of dies (°C)	20
Flanging and cooling (3rd to 5th stages)	Heat transfer coefficient between workpiece and air (W⋅m^−2^⋅K^−1^)	10
Whole process (1st to 5th stages)	Temperature of room *T*_room_ (°C)	20

**Table 4:** Comparison of geometrical parameters between FEM and experiment.

		*D_x_* (mm)	*D_z_* (mm)	*H_y_* (mm)
Experimental results		790	782	1088
FEM results	After flanging	786.707	784.716	1091.181
	After springback	787.434	784.214	1090.919
	After cooling	787.496	784.180	1090.657
